# Meibomian gland epithelial cells demonstrate enhanced reliance on glycolysis for energy metabolism and differentiation

**DOI:** 10.1186/s40662-026-00518-9

**Published:** 2026-11-01

**Authors:** Yaoyao Ren, Wenjing Li, Yuan Zhang, Yueli Tian, Shiya Yi, Xueling Tian, Yuehui Chen, Min Ke, Fan Xia, Yang Liu

**Affiliations:** 1 Department of Ophthalmology, Zhongnan Hospital of Wuhan University, Wuhan, 430071 Hubei China; 2 Department of Nuclear Medicine, Zhongnan Hospital of Wuhan University, Wuhan, China; 3 Department of Ophthalmology and Visual Sciences, University of Texas Medical Branch, Galveston, TX 77555 USA

**Keywords:** Meibomian gland, Glycolysis, Hypoxia, PET-CT

## Abstract

**Background:**

Meibomian gland dysfunction is a leading cause of dry eye disease. Previous studies have established that the meibomian gland exists in a physiological hypoxic environment, with hypoxia-inducible factor 1α playing a regulatory role. However, the metabolic strategy of this gland remains unclear. This study aimed to test the hypothesis that glycolysis serves as an important metabolic pathway in meibomian gland epithelial cells in vivo and that these cells have an enhanced glycolytic phenotype during differentiation.

**Methods:**

We combined clinical imaging, tissue analysis, and in vitro experiments to test our hypothesis.

**Results:**

^18^F-fluorodeoxyglucose positron emission tomography/computed tomography revealed significantly higher glucose uptake in human meibomian glands than in the orbicularis oculi muscle and surrounding subcutaneous connective tissues outside the tarsal plate, indicating substantial glycolytic activity in vivo. Immunofluorescence staining confirmed strong expression of glycolytic markers, including hypoxia-inducible factor 1α, glucose transporter type 1, hexokinase 2, 6-phosphofructo-2-kinase/fructose−2,6-biphosphatase 3, and pyruvate kinase M2, and lactate dehydrogenase A, in both human and mouse meibomian gland tissues. In immortalized human meibomian gland epithelial cells, differentiation induced a metabolic shift favoring glycolysis with increased extracellular acidification rate, elevated lactate production, and upregulated glycolytic enzyme expression. Three-dimensional spheroid cultures further revealed state-dependent metabolic heterogeneity.

**Conclusion:**

The findings indicate that meibomian gland epithelial cells have a highly glycolytic phenotype in vivo and markedly enhanced glycolytic dependence during differentiation. These support their specialized secretory functions and adaptation to hypoxic microenvironments.

**Supplementary Information:**

The online version contains supplementary material available at https://doi.org/10.1186/s40662-026-00518-9.

## Background

Meibomian glands are specialized sebaceous glands located within the eyelids and are responsible for secreting lipids that form a crucial part of the tear film. These secretions help maintain ocular surface lubrication and prevent excessive tear evaporation. Meibomian gland dysfunction (MGD) causes tear film instability and dry eye disease. According to the TFOS DEWS III report, MGD affects approximately 35.8% of the global population and imposes a substantial clinical and socioeconomic burden worldwide [1]. Our previous studies have shown that meibomian glands exist in a physiological hypoxic environment, and hypoxia-inducible factor 1α (HIF-1α) plays a critical role in the regulation of the gland [2, 3]. These results make sense, because several key aspects of meibomian gland structure support the maintenance of a low O_2_ environment: (i) the meibomian gland lacks intraglandular blood flow, as its vasculature lies outside the basement membrane, limiting oxygen diffusion into the gland [4]; (ii) differentiating meibocytes migrate toward the acinar center, increasing the distance from the peripheral oxygen source; and (iii) meibomian gland acini measure approximately 150–200 μm in diameter [4], which is at the upper limit of oxygen diffusion capacity (~ 150 μm) [5]. Together, these features suggest the presence of an oxygen gradient within the gland, with the lowest O₂ concentration in the central acinar region where holocrine secretion occurs. This raises the following fundamental questions: How do meibomian gland cells metabolize under hypoxic conditions? Do basal proliferating cells differ metabolically from central differentiating cells within the acini?

Oxidative phosphorylation (OXPHOS) and glycolysis are the two principal adenosine triphosphate (ATP)-generating pathways involved in cellular metabolism. OXPHOS predominates under aerobic conditions and produces the majority of cellular ATP. In contrast, glycolysis generates ATP rapidly and independent of oxygen, making it essential for survival in anaerobic and hypoxic environments. Although glycolysis yields less ATP per glucose molecule than OXPHOS, it provides critical intermediates (e.g., glyceraldehyde−3-phosphate and pyruvate) that fuel biosynthetic processes such as lipid synthesis [6]. Critically, under hypoxia, HIF-1α stabilization activates genes encoding glycolytic enzymes and glucose transporters (e.g., glucose transporter type 1 [GLUT1]), thereby enhancing glucose uptake and lactate production to sustain ATP generation through glycolysis [7]. Based on our previous identification of the meibomian gland as a physiologically hypoxic tissue, we hypothesized that the meibomian gland would demonstrate a highly glycolytic phenotype in vivo.

To test this hypothesis, we conducted a clinical study using ^18^F-fluorodeoxyglucose (^18^F-FDG) positron emission tomography (PET), a functional imaging technique that detects tissue metabolic activity [8]. The radiotracer ^18^F-FDG, a glucose analog, is transported into cells primarily via glucose transporters (e.g., GLUT1) and is phosphorylated by hexokinase. The phosphorylated product, FDG-6-phosphate, is not a substrate for the subsequent enzyme in glycolysis. Unlike glucose-6-phosphate, it cannot be further metabolized via glycolysis or enter the tricarboxylic acid cycle, which leads to metabolic trapping within the cell. Consequently, the rate of FDG accumulation is directly proportional to the rate of glucose entry into the glycolytic pathway, enabling sensitive detection of tissues with high glycolytic flux observed in both pathological (e.g., tumors) and physiological (e.g., active muscle) contexts. Thus, ^18^F-FDG PET provides a powerful approach for investigating glycolytic metabolism in the meibomian gland in vivo.

Furthermore, we examined glycolytic pathway markers in human and mouse meibomian gland tissue sections and evaluated the metabolic status of cultured human meibomian gland epithelial cells under various conditions. Taken together, these studies aimed to test our hypothesis that glycolysis is a major metabolic pathway supporting meibomian gland function.

## Methods

### Patient information

We retrospectively analyzed data from 70 patients who underwent clinical ^18^F-FDG PET/CT for unrelated indications at the Zhongnan Hospital of Wuhan University between January 1, 2023, and December 31, 2024. The inclusion criteria were as follows: (a) age 18–80 years; (b) no history of ocular trauma or ocular surgery; (c) at least one completed ^18^F-FDG PET/CT scan. The exclusion criteria were as follows: (a) incomplete key medical records required for analysis; (b) concurrent participation in other interventional trials; (c) confirmed eyelid tumors. This retrospective study was conducted in accordance with the Declaration of Helsinki and approved by the Medical Ethics Committee of Zhongnan Hospital of Wuhan University (Approval No. 2024026 K). As this was a retrospective study without patient intervention or tissue collection, the requirement for individual informed consent was waived by the ethics committee.

Human meibomian gland tissues and primary human conjunctival fibroblasts were obtained from donated cadavers at the Zhongnan Hospital of Wuhan University. Written informed consent was obtained from immediate family members in strict accordance with the Declaration of Helsinki and approved by the Medical Ethical Committee (No. ZN2025212K).

### Quantification of glycolytic activity via PET/CT

Glycolytic activity within the meibomian glands was quantified using standardized uptake values (SUVs) derived from fused ^18^F-FDG PET/CT images. The 10 mm spherical regions of interest (ROIs) were centered precisely on the tarsal plates of the upper and lower eyelids and identified via high-resolution sagittal and axial CT fusion images. Adjacent muscle and subcutaneous tissues outside the orbital rim were used as internal background controls. We used the maximum standardized uptake value (SUVmax) because it represented the single pixel with the highest radiotracer concentration within the ROI. In small organs prone to partial-volume effects, SUVmean is heavily diluted by the surrounding inactive or low-uptake tissues (such as the palpebral conjunctiva or skin). Therefore, although SUVmax may be more susceptible to image noise than SUVmean, it is less affected by dilution from surrounding low-uptake tissues and has been widely used to assess focal tracer accumulation in small anatomical structures. Given the small size of the meibomian glands, we considered the SUVmax to be the most appropriate metric for exploratory analysis. Retrospective image analysis was performed independently by two nuclear medicine specialists who were blinded to the patients’ clinical ophthalmic history. Paired t-tests were used to compare the SUVmax between meibomian glands and adjacent tissues in each patient. An elevated SUVmax indicates enhanced glycolytic flux, directly testing whether meibomian glands display high glycolytic activity consistent with their hypoxic physiology.

### Animals

All animal procedures were approved by the Animal Ethics Committee of the Zhongnan Hospital of Wuhan University (Approval No. ZN2024064) and performed in compliance with the ARRIVE guidelines. SPF-grade C57BL/6 mice were housed under specific pathogen-free conditions (temperature: 22 ± 1 °C, humidity: 55 ± 10%, 12-h light/dark cycle) with ad libitum access to food and water. All efforts were made to minimize animal suffering and to reduce the number of animals used. Mice were euthanized by CO_2_ asphyxiation in accordance with the AVMA Guidelines for Euthanasia of Animals.

### Cell culture

Immortalized human meibomian gland epithelial cells (IHMGECs; ATCC, CRL-3472) were maintained in Keratinocyte-SFM medium (Thermo Fisher Scientific, 17,005,042) supplemented with 5 ng/mL recombinant human epidermal growth factor and 50 μg/mL bovine pituitary extract. Cell differentiation was induced by culturing the cells for 7 days in DMEM/F-12 (1:1; Cytiva) supplemented with 10% fetal bovine serum (FBS; Cytiva) [9]. Primary human conjunctival fibroblasts were kindly provided by Dr. Ming Yan of the Zhongnan Hospital of Wuhan University (China). This study involving primary human conjunctival fibroblasts was conducted in accordance with the Declaration of Helsinki and approved by the Medical Ethics Committee of Zhongnan Hospital of Wuhan University (Approval No. 2025212 K). Written informed consent was obtained from all the participants. The HeLa cells were provided by Dr. Le Liang of the Institute for Advanced Studies, Wuhan University (China). All the cells were incubated at 37 °C in 5% CO₂.

### Three-dimensional cell culture

Three-dimensional (3D) cultures of IHMGECs were performed using Matrigel as previously described [10]. IHMGECs were resuspended in ice-cold keratinocyte-SFM and mixed with Matrigel (Vazyme, Plurigel Matrix, LDEV-Free, GL101-01) on ice at a 4:1 (Matrigel:medium) ratio to achieve a final concentration of 5 × 10^4^ cells/mL. A 50 μL aliquot of the ice-cold cell-Matrigel suspension was added to each well of a pre-warmed 24-well plate. The plate was incubated at 37 °C with 5% CO₂ for 60 min to allow for Matrigel polymerization. After polymerization, keratinocyte SFM was added to each well. The cells were maintained under standard conditions (37 °C, 5% CO₂) to form spheroids. After two weeks, the proliferation medium was replaced with a differentiation medium. Spheroids were then collected separately from the proliferation and differentiation cultures.

### Metabolomics analysis

Non-targeted metabolomics was performed on IHMGECs in their proliferation and differentiation states, with three biological replicates per group. Metabolomic analysis was performed by Wuhan Metware Biotechnology Co., Ltd. using ultra-performance liquid chromatography-tandem mass spectrometry. Metabolites were extracted using a two-step protocol: hydrophilic compounds were extracted with 80% methanol and hydrophobic compounds were extracted with a methyl-tert-butyl ether/methanol system. Separation was achieved using T3 C18 (positive and negative ion modes), hydrophilic interaction liquid chromatography (negative ion mode), and C30 columns (for lipids), with detection performed in the multiple reaction monitoring mode. Metabolites were identified using in-house MetWare database and public databases (Kyoto Encyclopedia of Genes and Genomes and Human Metabolome Database). Data were normalized to protein concentrations and quality control samples were included to ensure reproducibility. The Orthogonal projections to latent structure-discriminant analysis model was validated for overfitting using 200-times permutation test. The resulting quality parameters (R^2^Y = 0.98 and Q^2^ = 0.91) indicated excellent model stability and highly reliable predictive capacity. The threshold for identifying differential metabolites was rigorously set at Variable Importance in Projection > 1.0, and a Benjamini–Hochberg adjusted *P* value of < 0.05.

### Seahorse metabolic analyzer assays

Cellular glycolytic rates were measured using an Agilent Seahorse XFe24 Analyzer (Agilent Technologies) and a Glycolytic Rate Assay Kit (Agilent Technologies, 103,344–100). Proliferating IHMGECs, differentiated IHMGECs, HeLa cells (i.e. glycolysis positive controls) [6, 11], and human primary conjunctival fibroblasts (i.e. OXPHOS positive controls) [12] were seeded at 4 × 10^4^ cells per well in XFe24 microplates (n = 5 independent cell culture biological replicates). Prior to analysis, sensor cartridges were hydrated overnight at 37 °C in non-CO₂ conditions using Seahorse XF Calibrant Solution. On the assay day, cell confluence and morphology were verified, followed by washing with pre-warmed assay medium and a 60-min incubation at 37 °C without CO₂. Immediately before measurement, medium was replaced with fresh assay medium, and inhibitors (0.5 μM rotenone/antimycin A in Port A; 50 mM 2-deoxy-D-glucose in Port B) were loaded into the calibrated sensor cartridge. Real-time extracellular acidification rate (ECAR) measurements were recorded after calibration and subsequent data analysis was performed using GraphPad Prism software. One-way ANOVA followed by Tukey’s post-hoc test was used for multigroup cellular comparisons. Normality and homogeneity of variance were assessed using Shapiro–Wilk and Brown–Forsythe tests, respectively.

### Lactate quantification

Lactate levels in IHMGECs were measured using a colorimetric Lactate Assay Kit (Cat # P0393S; Beyotime) based on the WST-8 reaction. Briefly, cells were washed with PBS and lysed on ice in BeyoLysis™ A buffer (200 μL per 1×10⁶ cells). Lysates were centrifuged at 12,000 × g for 5 min at 4 °C to pellet cellular debris, and the supernatants were collected. For the assay, the supernatants were incubated at 37 °C for 30 min with a freshly prepared WST-8 working solution (Lactate Assay Buffer, Enzyme Solution, Substrate Solution, and WST-8 Solution at 44:2:2:2). Absorbance was measured at 450 nm, and lactate concentrations were determined from a standard curve generated using serial dilutions of a 100 mM L-lactate standard.

### Immunofluorescence

Human meibomian gland tissues from donated cadavers were collected in accordance with the Declaration of Helsinki and approved by the Medical Ethics Committee of Zhongnan Hospital of Wuhan University (Approval No. 2025212 K). Written informed consent was obtained from all participants.. Human meibomian gland tissues, mouse meibomian gland tissues, and Matrigel-embedded spheroids were fixed in 4% paraformaldehyde (48 h, 4 °C), dehydrated using graded ethanol, cleared in xylene, and embedded in paraffin. Sections (4–6 μm) underwent heat-induced antigen retrieval in citrate/EDTA buffer, followed by blocking with 10% donkey serum (30 min, room temperature [RT]). Sections were incubated overnight at 4 °C with primary antibodies, then with Alexa Fluor® 488-conjugated secondary antibodies (1 h, RT, dark). Nuclei were counterstained with 4′,6-diamidino-2-phenylindole (1:500, 10 min, RT). Finally, the slides were mounted with Antifade Mounting Medium (Beyotime, P0131) and imaged using an Aperio VERSA 8 slide scanner (Leica Biosystems). The antibodies used are listed in Table 1. Fluorescence intensity was quantified using ImageJ software by measuring the integrated density of the meibomian gland acinar regions, normalized directly to the total area of the acinus, with background fluorescence from a non-tissue area subtracted from each slide to ensure parity.

**Table 1:** Antibodies used in the study

Antibody name	Dilution	Source	Catalog number	Application
anti-GLUT1	1:200 (IF) 1:1000 (WB)	Sigma	SAB4502803	IF; WB
anti-Hexokinase 2	1:500 (IF) 1:10,000 (WB)	Abcam	ab227198	IF; WB
anti-PKM2	1:100 (IF) 1:1000 (WB)	Abmart	T55764F	IF; WB
anti-PFKFB3	1:200 (IF) 1:1000 (WB)	Invitrogen	MA572806	IF; WB
anti-LDHA	1:100 (IF)	Abclonal	A1146	IF
anti-HIF-1α	1:200 (IF)	Santa Cruz	SC-13515	IF
anti-β-actin	1:30,000 (WB)	Abclonal	AC004	WB
Alexa Fluor® 488 donkey anti-rabbit IgG	1:500 (IF)	Abcam	ab150073	IF
Alexa Fluor® 488 donkey anti-mouse IgG	1:500 (IF)	Abcam	ab150105	IF
HRP-conjugated goat anti-rabbit IgG (H+L)	1:10,000 (WB)	Abcam	ab6721	WB
HRP-conjugated goat anti-mouse IgG (H+L)	1:10,000 (WB)	Abcam	ab6789	WB

GLUT1 = glucose transporter 1; H+L = heavy and light chains; HIF‑1α = hypoxia‑inducible factor‑1α; HRP = horseradish peroxidase; HK2 = hexokinase 2; IF = immunofluorescence; IgG = immunoglobulin G; LDHA = lactate dehydrogenase A; PFKFB3 = 6‑phosphofructo‑2‑kinase/fructose‑2,6‑bisphosphatase 3; PKM2 = pyruvate kinase M2; WB = Western blot

### H&E and Masson staining

H&E and Masson’s trichrome staining were performed to visualize the morphology of MG tissues. For H&E staining, the sections were first stained with hematoxylin to visualize the nuclei, followed by counterstaining with eosin to highlight the cytoplasmic and extracellular components. Masson’s trichrome staining was performed sequentially with hematoxylin for nuclei, ponceau acid fuchsin for the cytoplasm and muscle, and aniline blue for collagen.

### Western blot

Following PBS washes, the IHMGECs were lysed in RIPA lysis buffer (Beyotime; P0013B) supplemented with 1 mM PMSF (Servicebio; G2008) and a protease/phosphatase inhibitor cocktail (Servicebio; G2006). Lysates were centrifuged (12,000 × g, 10 min, 4 °C), and the supernatants were collected. Protein concentrations were determined using bicinchoninic acid assay (Beyotime; P0010S). Samples were diluted 1:4 in SDS loading buffer, boiled for 7 min, resolved by SDS-PAGE, and transferred onto PVDF membranes. Membranes were blocked with 5% non-fat dry milk (2 h, RT) and incubated with primary antibodies (overnight, 4 °C), followed by HRP-conjugated secondary antibodies (2 h, RT). After washing with PBST, the bands were visualized using Omni-ECL (Epizyme, SQ203) and imaged using a Tanon 5200 system. Band intensities were quantified using ImageJ (v1.8.0), with β-actin serving as a loading control for normalization across all samples. The antibodies used are listed in Table 1.

## Results

### The meibomian gland has a glycolytic metabolic profile in vivo

**Fig. 1:**
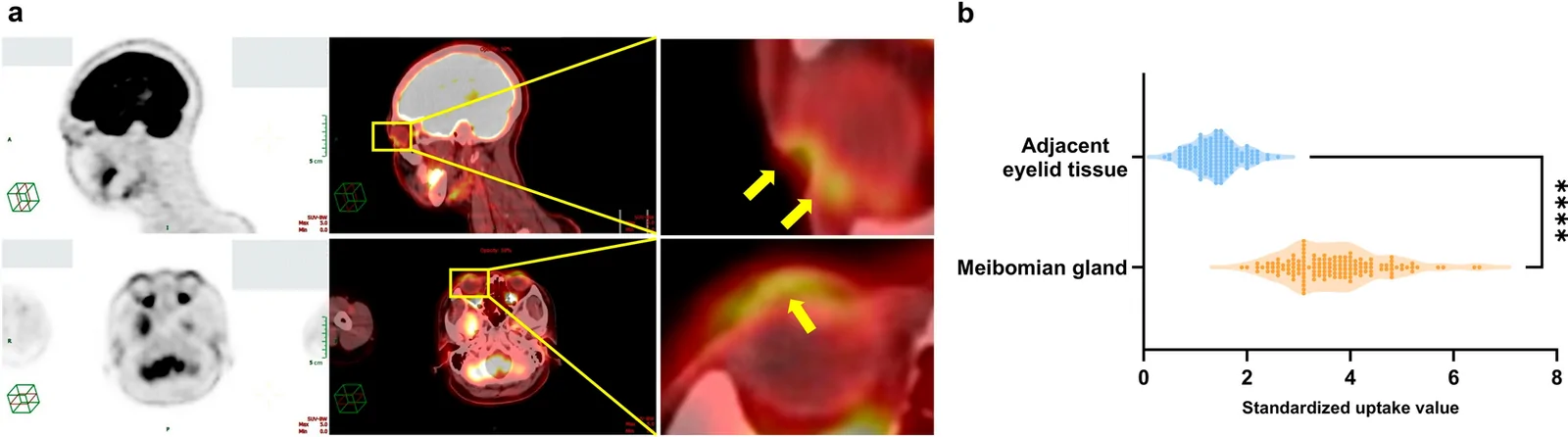
Elevated glucose uptake in meibomian glands on ^18^F-fluorodeoxyglucose (^18^F-FDG) positron emission tomography (PET)/computed tomography (CT).** a** Representative sagittal and axial ^18^F-FDG PET/CT images of patients are shown, including PET and fused PET/CT panels. Yellow boxes indicate magnified regions and yellow arrows highlight areas of prominent ^18^F-FDG accumulation in the meibomian glands. **b** Quantification of standardized uptake values of ^18^F-FDG in the meibomian glands, orbicularis oculi muscle, and surrounding subcutaneous connective tissues outside the tarsal plate, revealing significantly higher radiotracer uptake in the meibomian glands than in the adjacent tissues (****P < 0.0001, paired t-test; n = 70 unique patients for PET/CT)

To test the hypothesis that human meibomian gland tissues exhibit high glycolytic activity in vivo, we evaluated clinical FDG-PET/CT imaging data. The SUVmax values were compared between the meibomian glands, the orbicularis oculi muscle, and the surrounding subcutaneous connective tissues outside the tarsal plate within each patient. The results show that ^18^F-FDG PET imaging demonstrated significantly higher radiotracer uptake in the meibomian glands than in the adjacent tissues (*P* < 0.0001, paired t-test; Fig. 1). This pronounced signal intensity indicated substantial glucose utilization and glycolytic activity in vivo. Critically, the selective accumulation of ^18^F-FDG, which directly reflects the hexokinase-mediated trapping of phosphorylated FDG, confirms that meibomian glands have elevated glucose uptake and high glycolytic activity, which is consistent with their physiologically hypoxic state.

### In vivo expression of glycolytic markers in meibomian glands

**Fig. 2:**
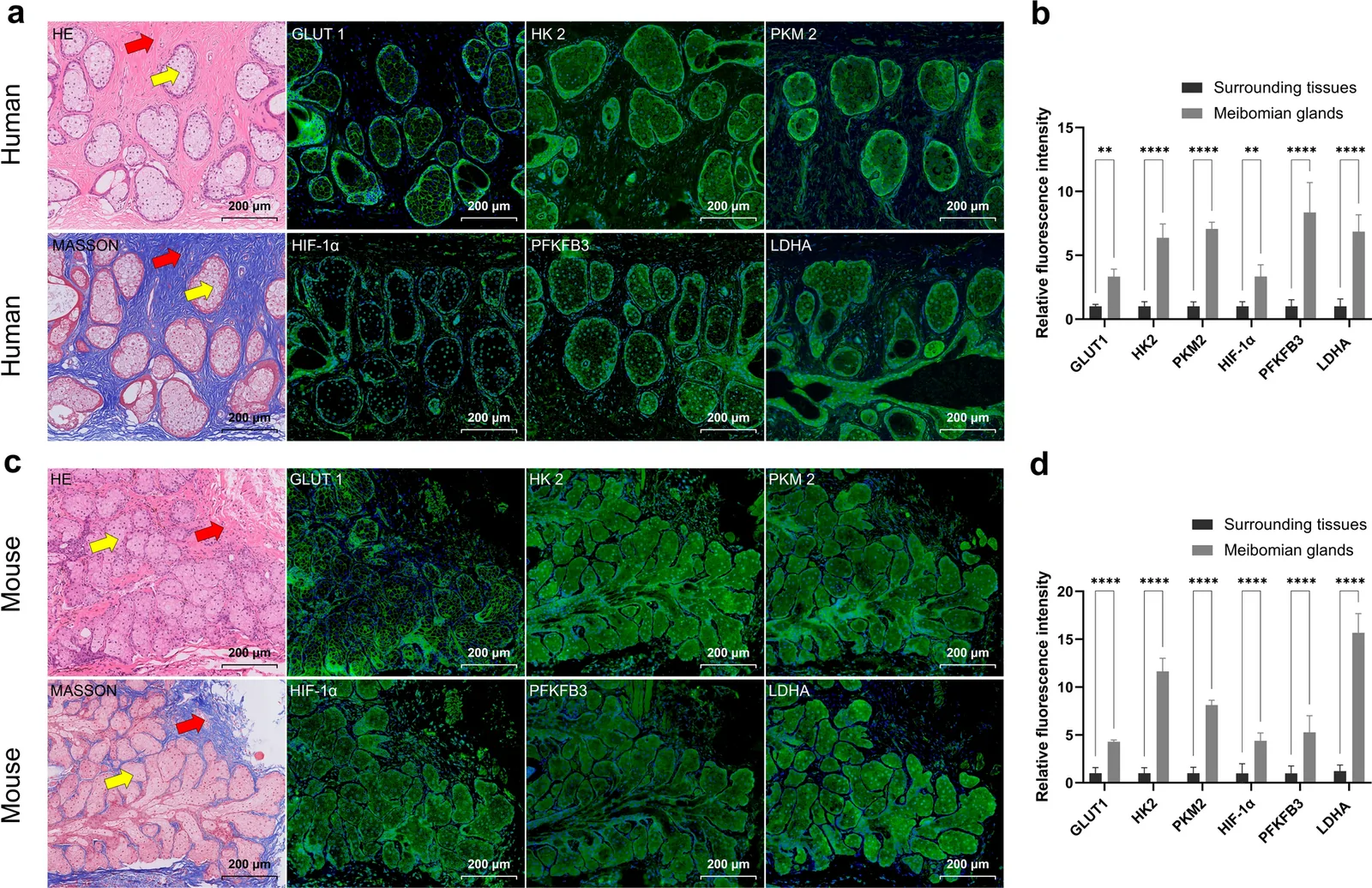
In vivo expression of glycolytic markers in meibomian glands.** a** Representative images of hematoxylin and eosin (HE), Masson, and immunofluorescence staining for hypoxia-inducible factor 1α (HIF-1α), glucose transporter 1 (GLUT1), hexokinase 2 (HK2), 6-phosphofructo-2-kinase/fructose−2,6-biphosphatase 3 (PFKFB3), pyruvate kinase M2 (PKM2), and lactate dehydrogenase A (LDHA) in meibomian gland tissues from humans. HE and Masson staining were performed to visualize the morphology of meibomian gland tissues. Green fluorescence indicates target protein expression and blue (4′,6-diamidino-2-phenylindole [DAPI]) staining indicates cell nuclei. Yellow arrows delineate the meibomian gland, and red arrows delineate the adjacent control tissues. **b** Quantification of glycolytic marker expression in human meibomian gland tissues. **c** Representative images of HE, Masson, and immunofluorescence staining for HIF-1α, GLUT1, HK2, PFKFB3, PKM2, and LDHA in meibomian gland tissues from mice. **d** Quantification of glycolytic marker expression in mouse meibomian gland tissues. (**P < 0.01, ****P < 0.0001, two-way ANOVA, n = 5 independent human cadavers and n = 5 independent mouse clusters)

To confirm the glycolytic phenotype of the meibomian gland in vivo, immunofluorescence staining of mouse and human meibomian glands revealed strong expression of HIF-1α and key glycolysis-related proteins, including GLUT1, hexokinase 2 (HK2), 6-phosphofructo-2-kinase/fructose−2,6-biphosphatase 3 (PFKFB3), and pyruvate kinase M2 (PKM2), and lactate dehydrogenase A (LDHA). These proteins were expressed at significantly higher levels in meibomian glands than in the surrounding tissues, supporting the presence of an active glycolytic metabolic profile in vivo (Fig. 2).

### Meibomian gland epithelial cells undergo a metabolic shift favoring glycolysis from proliferation to differentiation

To demonstrate that meibomian gland epithelial cells undergo a metabolic shift favoring glycolysis during the transition from proliferation to differentiation, cellular metabolites, metabolic activity, and key glycolytic enzymes were analyzed in both proliferating and differentiated cells using multiple assays.

**Fig. 3:**
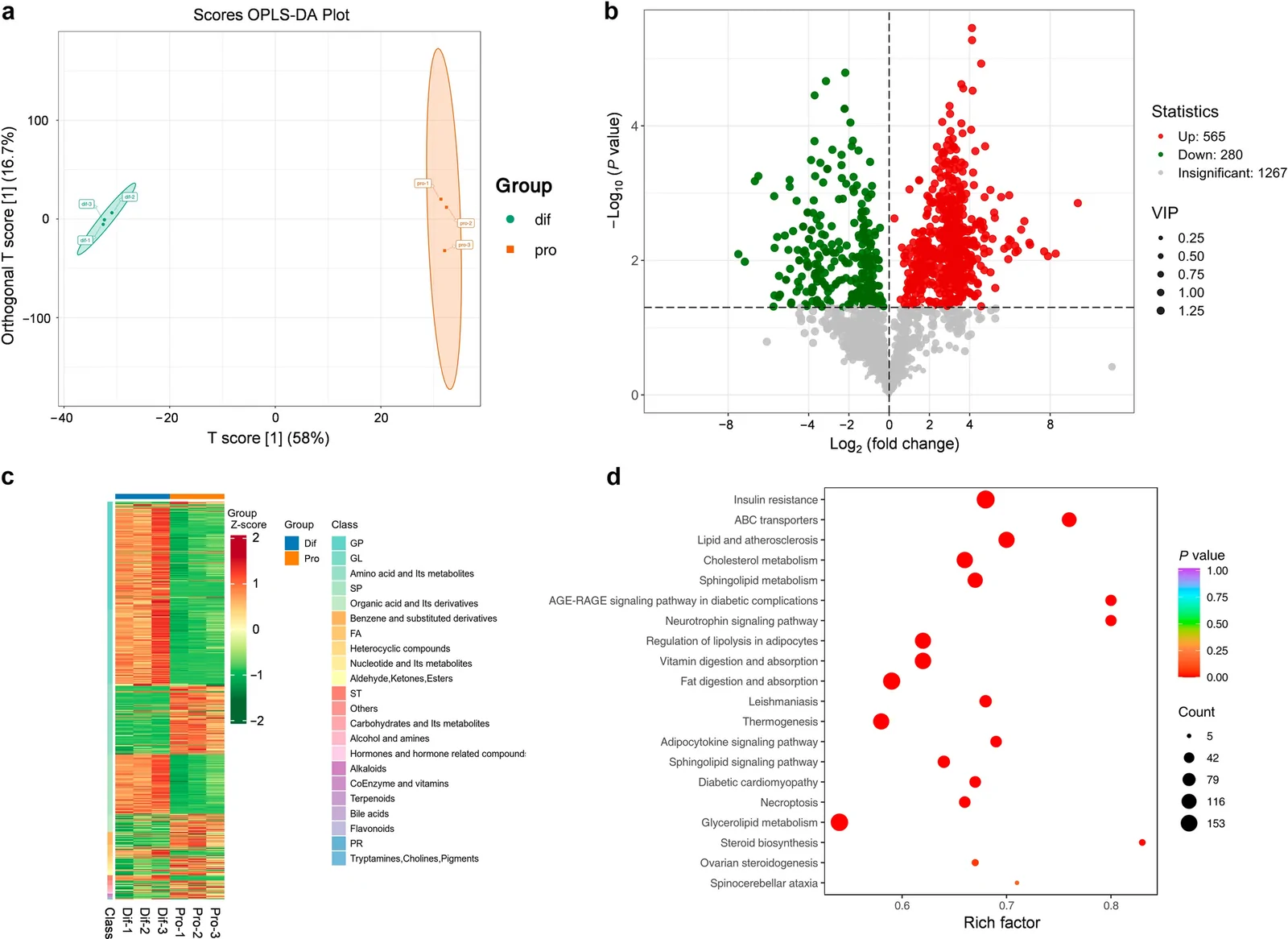
Non-targeted metabolomics on cells in proliferation and differentiation states.** a** Orthogonal projections to latent structures-discriminant analysis (OPLS-DA) score plot shows clear separation between proliferating (pro) and differentiated (dif) groups with tight intra-group clustering. The predictive component (x-axis, 58% variance explained) demonstrates strong discriminative power for differential metabolite analysis. **b** Volcano plot identifies 845 differential metabolites (565 upregulated, 280 downregulated in dif vs. pro) via variable importance in projection (VIP) > 1 and *P* value < 0.05. **c** Differential metabolite clustering heatmap displays ultraviolet-scaled relative abundances (red = high, green = low). Sample clustering (upper dendrogram) indicates strong intra-group consistency, while metabolite clustering (left dendrogram) reveals co-regulated metabolite patterns. **d** Kyoto encyclopedia of genes and genomes (KEGG) pathway enrichment plot links differential metabolites to key metabolic pathways. Rich factor (x-axis) and *P* value (dot color, red = more significant) indicate pathways significantly enriched

Non-targeted metabolomic analysis revealed marked differences between proliferating and differentiated IHMGECs. Orthogonal projections to latent structure-discriminant analysis score plots demonstrated clear separation of the metabolic profiles (Fig. 3a). Volcano plot analysis identified 845 significantly altered metabolites (565 upregulated and 280 downregulated in differentiated cells; Fig. 3b), and heatmap clustering showed consistent intragroup accumulation patterns (Fig. 3c). Kyoto encyclopedia of genes and genomes pathway enrichment analysis of these differential metabolites further mapped the global functional transitions that drive meibocyte maturation (Fig. 3d). Specifically, in the proliferating state, glucose utilization is predominantly channeled toward pathways that support cellular expansion and biomass accumulation. Conversely, upon terminal differentiation, the cellular metabolic network undergoes a distinct functional shift, routing carbon pathways away from simple biomass replication, and scaling up pathways dedicated to organic acid production and lipid backbone synthesis. This structural realignment of metabolic pathways provides the biosynthetic precursors and reducing equivalents required to sustain specialized lipid secretion and meibum production. Although non-targeted metabolomics demonstrated a distinct separation of global metabolic profiles between proliferating and differentiated IHMGECs, the steady-state levels of upstream glycolytic intermediates did not reach statistical significance (Supplementary Fig. 1). Therefore, to evaluate glycolytic activity, we performed dynamic extracellular flux analysis and precise quantification of terminal metabolites.

**Fig. 4:**
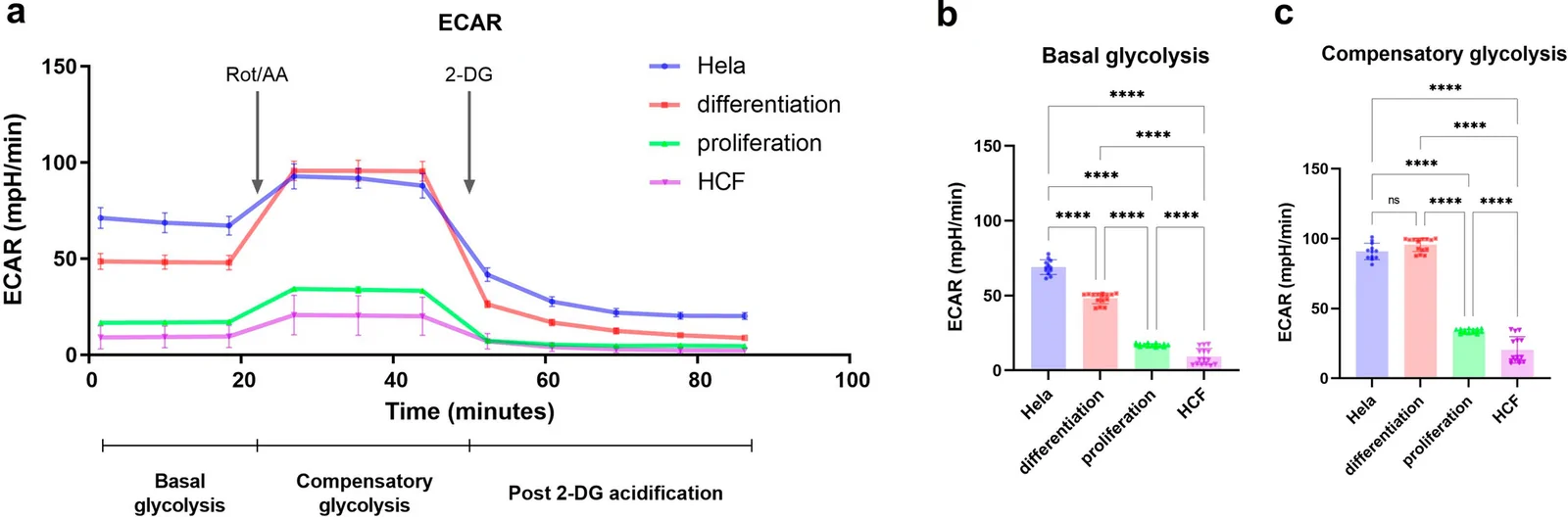
Glycolytic metabolic features of immortalized human meibomian gland epithelial cells (IHMGECs).** a** The extracellular acidification rate (ECAR, mpH/min) was measured using the Seahorse assay for four cell types: HeLa cells, differentiated IHMGECs, proliferating IHMGECs, and human conjunctival fibroblasts (HCFs). **b** Quantification of basal glycolysis in the four cell types. **c** Quantification of compensatory glycolysis in the four cell types. IHMGECs exhibited higher basal and compensatory glycolytic activity than HCFs, but lower basal glycolysis than HeLa cells. Differentiated IHMGECs show significantly higher basal and compensatory glycolytic rates than proliferating IHMGECs. (****P < 0.0001, n = 5 independent cell culture replicates; one-way ANOVA)

An Agilent Seahorse XFe24 Analyzer with the Glycolytic Rate Assay was used to measure basal and compensatory glycolytic rates in IHMGECs under various culture conditions. Meibomian gland epithelial cells demonstrate higher basal and compensatory glycolytic activities than human conjunctival fibroblasts, which primarily use OXPHOS as a major metabolic pathway. However, their basal glycolysis was lower than that of highly metabolically active HeLa tumor cells, which predominantly utilize glycolysis. Importantly, differentiated IHMGECs showed significantly higher activity of both basal and compensatory glycolysis than proliferating IHMGECs (Fig. 4).

**Fig. 5:**
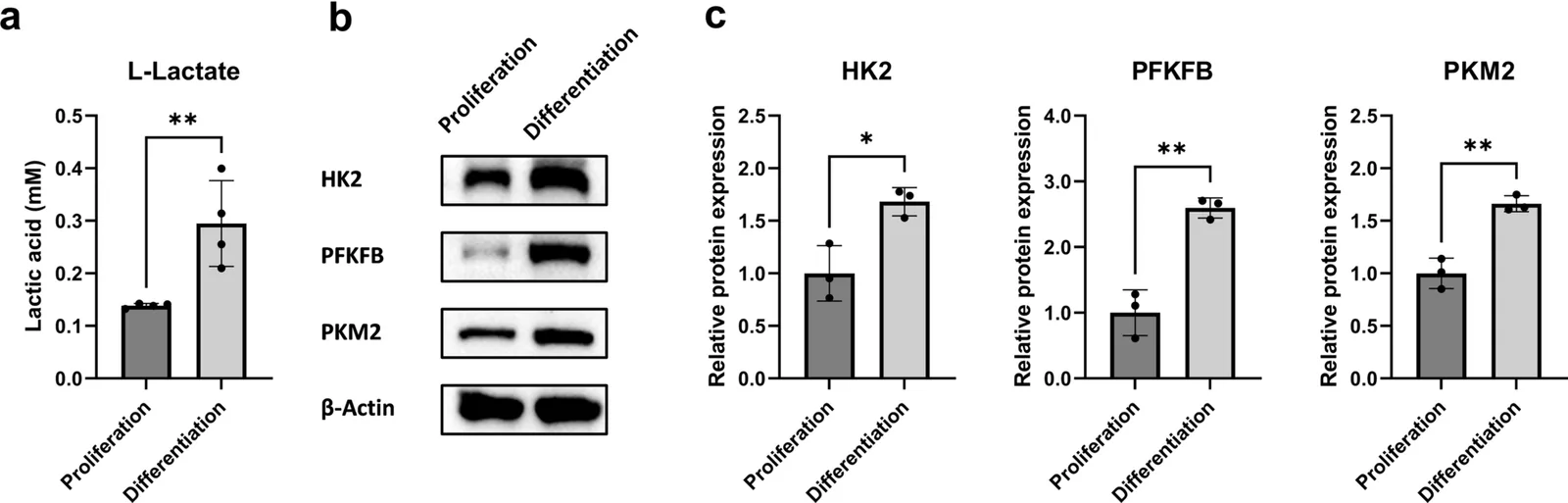
Glycolytic metabolic shift during immortalized human meibomian gland epithelial cells (IHMGECs) differentiation.** a** Lactic acid concentration (mM) in proliferating and differentiated IHMGECs. Differentiated cells had significantly higher lactate concentrations than proliferating cells (**P < 0.01, unpaired t-test; n = 4 independent cell culture biological replicates). **b** Representative Western blot images showing protein expressions of hexokinase 2 (HK2), 6-phosphofructo-2-kinase/fructose−2,6-biphosphatase 3 (PFKFB3), and pyruvate kinase M2 (PKM2), and β-actin in proliferating and differentiated IHMGECs. **c** Quantitative analysis of relative protein expression levels of HK2, PFKFB3, and PKM2 in proliferating and differentiated IHMGECs. Differentiated IHMGECs had increased expressions of HK2 (*P < 0.05), PFKFB3 (**P < 0.01), and PKM2 (**P < 0.01) compared with proliferating cells (unpaired t-test; n = 3 independent cell culture biological replicates). These results indicate a glycolytic metabolic shift during IHMGECs differentiation

These two key metrics comprehensively reflect the basal glycolytic activity and stress-responsive glycolytic reserves of the cells. Consistent with these findings, lactate production, the terminal metabolite of glycolysis [13], was significantly higher in differentiated cells than in proliferating cells (*P* < 0.01, n = 4 independent cell culture biological replicates; Fig. 5a). Western blot analysis further confirmed the significant upregulation of key glycolytic enzymes, including HK2, PFKFB3, and PKM2, in differentiated cells (*P* < 0.05, n = 3 independent cell culture replicates, Fig. 5b). Upregulation of HIF-1α and GLUT1 was not shown, as these findings have been demonstrated in our previous publication [3]. These results indicated a clear metabolic shift toward glycolysis during cellular differentiation in IHMGECs.

**Fig. 6:**
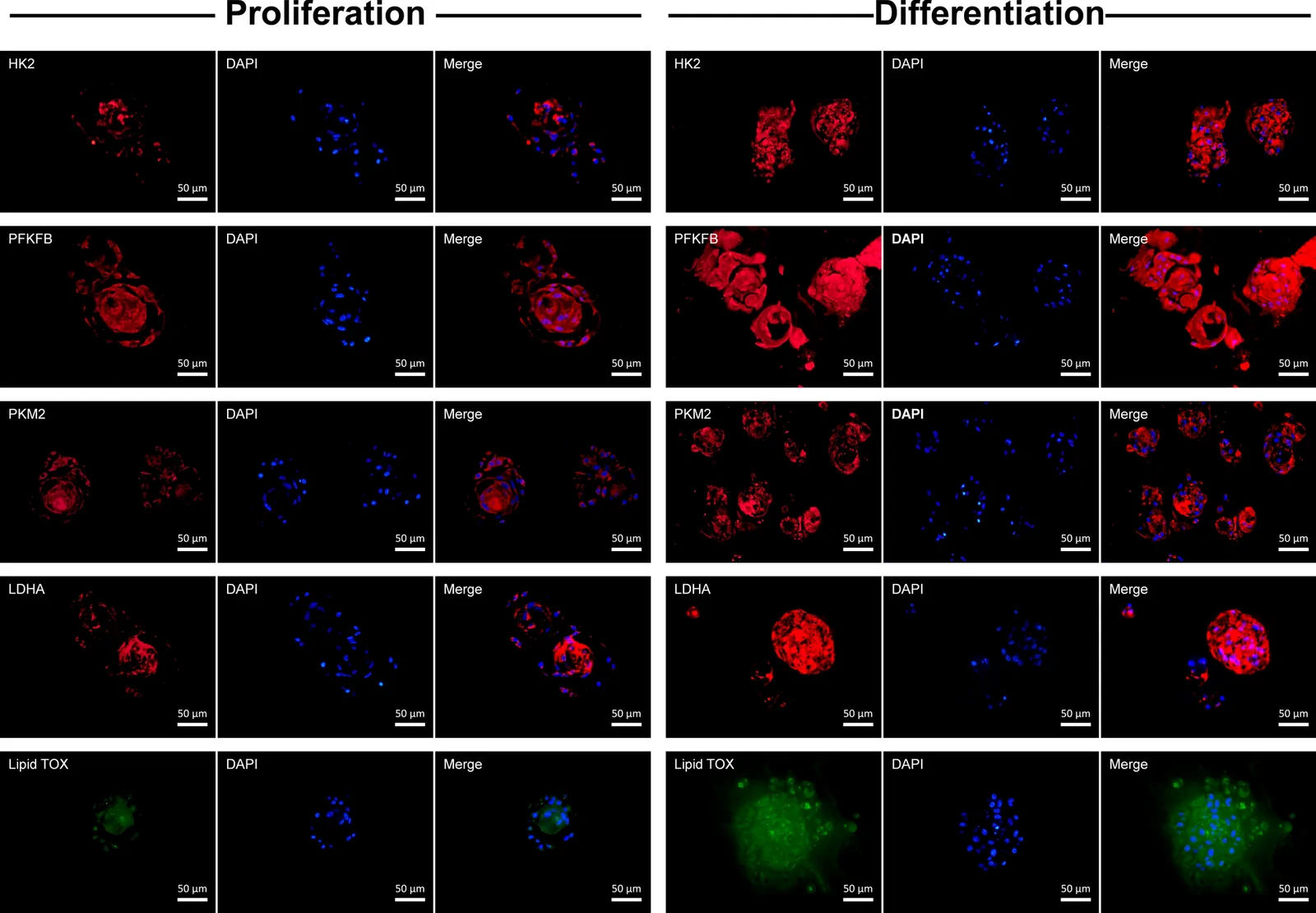
Three-dimensional cultured spheroids have state-dependent metabolic heterogeneity. Representative immunofluorescent images of spheroid sections stained for key glycolytic enzymes (hexokinase 2 [HK2], 6-phosphofructo-2-kinase/fructose−2,6-biphosphatase 3 [PFKFB3], pyruvate kinase M2 [PKM2], and lactate dehydrogenase A [LDHA]) and neutral lipids (LipidTOX, green). Nuclei were counterstained with 4′,6-diamidino-2-phenylindole (DAPI, blue)

To further validate these results, a 3D culture model was used to mimic in vivo conditions [10]. Immunofluorescence staining (Thermo Fisher Scientific, HCS LipidTOX, H34475) revealed a pronounced gradient in glycolysis-related protein and lipid expression in proliferative spheroids, with higher levels in the core regions. In contrast, differentiated spheroids exhibited a more uniform distribution of these markers (Fig. 6).

Collectively, these findings demonstrate that IHMGECs undergo a significant metabolic shift favoring glycolysis during differentiation, which is characterized by enhanced glycolytic activity and upregulation of key glycolytic enzymes.

## Discussion

Our study indicates that meibomian gland epithelial cells demonstrate a highly glycolytic phenotype both in vivo and in vitro, thereby supporting our hypothesis. Meibomian gland epithelial cells undergo a metabolic shift that favors glycolysis during differentiation.

This finding was demonstrated in living tissue through ^18^F-FDG PET imaging, a technique commonly used in oncology to detect tumors that exhibit intense radiotracer uptake due to their glycolytic phenotype (i.e., the Warburg effect). Notably, while malignant cells display elevated ^18^F-FDG signals due to preferential glycolysis even in the presence of oxygen, certain normal tissues with high metabolic demands (e.g., brain, myocardium) also exhibit physiological uptake. The significant ^18^F-FDG accumulation observed in meibomian glands places them among these glycolytically active physiological tissues, reflecting both high glucose uptake and metabolic trapping characteristic of glycolysis-dependent energy production.

Consistent with this observation, immunofluorescence staining confirmed the strong expression of glycolysis-related proteins in both human and mouse meibomian gland tissues compared to that in adjacent tissues. These findings further support our previous discovery that the meibomian gland functions as a physiologically hypoxic tissue, consistent with the nuclear localization of HIF-1α within the acinar cells.

A key finding was that meibomian gland epithelial cells exhibit distinct metabolic profiles depending on their cellular state and undergo a metabolic shift favoring glycolysis during the transition from proliferation to differentiation. Proliferating cells utilize mixed metabolic pathways, whereas differentiated cells exhibit substantial glycolytic activity. Metabolic shifts represent the adaptive restructuring of metabolic pathways to meet changing energy and biosynthetic demands [14]. Proliferating cells primarily support cell division and growth, whereas differentiated cells often require rapid ATP production to support specialized functions, of which glycolysis is the key metabolic pathway [14]. Notably, the differentiated cells were first maintained in proliferation medium until 70%–80% confluent and then switched to differentiation medium following a published protocol [9]. This change in culture conditions induced a cell fate transition accompanied by clear evidence of a metabolic shift, including increased ECAR, elevated expression of glycolytic markers, and enhanced lactate production. The in vitro differentiation protocol requires transitioning from a serum-free medium to serum-supplemented DMEM/F-12. Although this medium switch is necessary to initiate lipid synthesis and mimic terminal differentiation, serum components may independently modulate metabolic enzymes. Although our 3D culture system suggests an intrinsic metabolic response to microenvironmental gradients, future studies utilizing chemically defined single-variable differentiation factors are warranted to decouple environmental metabolic inputs from hardwired cell differentiation programs.

ECAR, a key indicator of glycolytic flux, was measured using a Seahorse metabolic analyzer. ECAR primarily reflects the efflux of lactic acid and protons into the extracellular environment during glycolysis [15]. In this study, basal and compensatory glycolysis were assessed. Basal glycolysis represents intrinsic glycolytic activity that maintains cellular homeostasis under steady-state conditions without external metabolic stress, whereas compensatory glycolysis reflects the maximum glycolytic capacity following mitochondrial inhibition by oligomycin. Oligomycin, an inhibitor of ATP synthase, blocks OXPHOS and forces cells to increase glycolysis to compensate for the loss of ATP production from the mitochondria [16]. Both proliferating and differentiated meibomian gland epithelial cells demonstrate a metabolic preference for glycolysis in vitro. Differentiated IHMGECs showed a significantly higher ECAR than proliferating cells, indicating an increased reliance on glycolysis. The basal glycolytic activity of meibomian gland epithelial cells in both states was lower than that of HeLa tumor cells but markedly higher than that of human conjunctival fibroblasts. Following oligomycin treatment, both proliferating and differentiated IHMGECs displayed a robust increase in compensatory glycolysis, demonstrating their metabolic flexibility in response to OXPHOS inhibition. Notably, the compensatory glycolytic rate of differentiated IHMGECs was comparable to that of HeLa cells, whereas proliferating IHMGECs exhibited lower compensatory glycolysis than differentiated cells but higher than fibroblasts. These findings provide a comprehensive understanding of enhanced glycolytic capacity associated with meibomian gland epithelial cell differentiation. Human meibomian gland epithelial cells demonstrate a significantly higher basal glycolytic rate and capacity than the adjacent human conjunctival fibroblasts under normoxia. This suggests that meibocytes are "hardwired" to favor glycolysis. When exposed to severe atmospheric hypoxia, fibroblasts upregulate glycolysis as a stress response, while meibomian gland epithelial cells maintain a sustained high-level glycolytic throughput, showing an evolutionarily adapted metabolic phenotype. We acknowledge the absence of direct oxygen consumption rate measurements in this study, which limits our ability to quantify the relative ATP contributions from glycolysis versus OXPHOS. However, our conclusion that differentiated IHMGECs exhibit an enhanced glycolytic phenotype is supported by multiple independent lines of evidence, including elevated lactate production, upregulation of glycolytic enzymes, and in vivo FDG‑PET/CT uptake. Future studies incorporating oxygen consumption rate analyses are warranted to fully delineate bioenergetic balance. While IHMGECs are invaluable, immortalization can inherently upregulate baseline glycolysis, and caution should be exercised when extrapolating absolute rates directly to in vivo tissues.

The significantly upregulated expression of key proteins in glycolysis pathways, including HIF-1α, GLUT1, HK2, PFKFB3 and PKM2 are consistent with the glycolytic characteristic and the physiological hypoxic nature of meibomian gland [17–21]. In addition to the total protein abundance, upregulated glycolytic enzymes can dynamically assemble into multi-enzyme complexes known as 'glycolytic metabolons' under hypoxic stress. This physical organization enables substrate channeling and increases metabolic efficiency. In the physiologically hypoxic environment of the meibomian gland where HIF-1α is stably expressed, the potential assembly of these metabolons could serve as a crucial adaptation to optimize ATP and biosynthetic output. Although capturing these macromolecular complexes was beyond the scope of this initial study, investigating their spatial configurations offers a new direction for elucidating MGD pathogenesis [22, 23]. Upstream pathways such as the mammalian target of rapamycin pathway can actively drive HIF-1α translation and glycolytic upregulation even in an oxygen-independent manner during cellular hypertrophy. Additionally, non-HIF pathways, such as the activation of AMP-activated protein kinase due to energy stress or the lipid-regulated peroxisome proliferator-activated receptor gamma axis, likely cooperate to modulate glucose transporter expression and coordinate the metabolic shift required for holocrine secretion.

The increased lactate production observed in differentiated cells is particularly noteworthy. Lactate, the end product of glycolysis, reflects enhanced glycolytic flux and supports specialized cellular activities such as lipid secretion. Increased lactate production in differentiated cells suggests that these cells are metabolically active and are likely to support specialized secretory functions, such as lipid secretion. This result is consistent with our previous finding that the pH of the cytoplasm decreases significantly during cell differentiation [2, 3]. A decrease in cytoplasmic pH is critical during meibomian gland differentiation because cytoplasmic and lysosomal acidification are interconnected [24, 25]. The meibomian gland holocrine system is a unique and destructive DNase II-dependent form of programmed cell death that involves complete cellular disintegration [26]. DNase II, a lysosomal endonuclease that degrades DNA, exhibits pH-sensitive activity peaking under acidic conditions (pH 4.5–5.5) [27, 28]. Within meibomian gland epithelial cells, an acidic lysosomal environment promotes DNase II activation. Activated DNase II translocates from the lysosomes to the nucleus to initiate a DNase II-dependent cell death pathway [26, 29]. We previously demonstrated that DNase II expression is exclusive to lipid-containing differentiated meibomian gland cells and significantly increases with the progression of differentiation [2, 30]. These findings suggest a potential mechanism whereby elevated lactate production contributes to sustaining the acidic intracellular microenvironment required for DNase II activation. This conceptual link provides an attractive hypothesis for how the metabolic shift aligns with holocrine secretory death; however, direct genetic or pharmacological inhibition of this pathway is required to confirm this causal sequence. Accelerated glycolysis yields an abundance of pyruvate that enters the mitochondria to generate acetyl-CoA via the pyruvate dehydrogenase complex. Acetyl-CoA is subsequently exported back into the cytoplasm to serve as a foundational building block for fatty acid elongation and cholesterol synthesis, which are the primary components of meibum. Furthermore, routing glucose through the auxiliary pentose phosphate pathway provides the nicotinamide adenine dinucleotide phosphate -reducing equivalents necessary to drive these highly endergonic lipogenic pathways. This explains why glycolytic scaling is functionally hardwired during terminal meibocyte differentiation.

In this study, while our functional kinetic assays (i.e., Seahorse ECAR) and terminal product analyses (i.e., lactate quantification) consistently demonstrated accelerated glycolysis in differentiated IHMGECs, the steady-state levels of several upstream glycolytic intermediates, such as glucose-6-phosphate, fructose-6-phosphate, and pyruvate, did not achieve uniform statistical significance (Supplementary Fig. 1). This apparent discrepancy highlights an inherent limitation of static, nontargeted metabolomics, which measures net metabolite pool sizes rather than dynamic metabolic flux. In highly active secretory cells like differentiating meibocytes, upstream intermediate metabolites are often rapidly consumed and channeled into downstream biochemical pathways, such as Acetyl-CoA generation for lipid synthesis and nicotinamide adenine dinucleotide phosphate production via the pentose phosphate pathway, as fast as they are generated. Consequently, accelerated glycolytic throughput can occur along with relatively unchanged or fluctuating steady-state intermediate levels. Therefore, functional real-time assessment of proton efflux and precise terminal metabolite capture are more accurate indicators of active glycolytic engagement during meibomian gland differentiation than static intracellular metabolomic profiling.

The 3D culture of IHMGECs provided further insight into the in vitro metabolic behavior, as the spheroid structure closely mimicked in vivo acinar organization. Oxygen diffusion in spheroids occurs from the surrounding Matrigel toward the center, highly consistent with established theoretical mathematical models of oxygen diffusion limitations in multicellular spheroids, where local hypoxia typically manifests at depths exceeding 100–150 μm. This spatial heterogeneity of glycolysis markers within the proliferative spheroids suggests that oxygen limitation is associated with a core-specific shift toward glycolytic metabolism and lipid accumulation. In contrast, differentiated spheroids exhibited uniformly high glycolytic activity, reflecting a metabolic shift that favors glycolysis during differentiation.

This shift is critical and consistent with our previous observations of significant gene expression changes during the proliferation-to-differentiation transition and the ability of cells to reversibly switch states in response to environmental cues [30, 31). However, the trigger that initiates differentiation and loss of proliferative capacity in meibomian gland cells remains elusive. Determining whether differentiated cells can undergo dedifferentiation and re-enter the cell cycle in vivo (e.g., via paligenosis) is crucial, as this potential holds the key to regenerating atrophied meibomian gland acini [31].

We acknowledge that the current findings primarily establish a strong correlation between meibocyte differentiation and enhanced glycolytic activity rather than direct causality. While our 3D spheroid model demonstrated that glycolytic markers naturally scale along spatial microenvironmental gradients, functional perturbation studies, such as pharmacological inhibition with 2-deoxy-D-glucose or targeted genetic knockdowns, will be required in future investigations to definitively establish the causal necessity of this pathway in meibomian gland homeostasis.

## Conclusions

In conclusion, understanding the metabolic distinctions between proliferative and differentiated meibomian gland epithelial cells is fundamental because regenerative capacity is inherently limited by metabolic constraints. Future studies are needed to investigate the regulatory mechanisms governing glycolysis in these cells and their potential therapeutic effects. This glycolytic metabolic strategy may enable the meibomian gland cells to sustain their functions in hypoxic environments and rapidly generate energy to support their secretory activities. Elucidating these metabolic processes is essential for advancing our understanding of meibomian gland physiology and identifying how metabolic dysregulation contributes to the pathogenesis of MGD.

From a translational perspective, our finding that the glycolytic phenotype is associated with normal meibocyte homeostasis provides novel therapeutic avenues. When meibomian glands undergo pathological changes, such as hyperkeratinization or ductal occlusion, the local physiological hypoxic microenvironment can be disrupted, leading to metabolic dysregulation, where meibocytes fail to scale up glycolysis. This metabolic failure directly initiates downstream lipogenesis, culminating in lipid-deficient MGD and severe evaporative dry eye. Consequently, targeting these pathways using local metabolic modulators is a highly promising therapeutic strategy for reactivating healthy glycolytic flux and restoring tear film lipid secretion. Specifically, small-molecule prolyl hydroxylase inhibitors, such as Roxadustat, which stabilize HIF-1α and are already clinically approved for systemic indications, could potentially be repurposed as topical ophthalmic formulations [2, 3]. By pharmacologically reinforcing the physiological hypoxic signaling cascade and promoting downstream glycolytic efficiency, these agents may help restore meibomian gland function in patients with severe MGD and evaporative dry eye.

## Supplementary Information

Supplementary Material 1

## Data Availability

No datasets were generated or analysed during the current study.
