# Supplementary material for: Meibomian gland epithelial cells demonstrate enhanced reliance on glycolysis for energy metabolism and differentiation
Source: Eye Vis (Lond). 2026 Nov 1;13:48. doi: 10.1186/s40662-026-00518-9 (PMC13723944; doi:10.1186/s40662-026-00518-9)
Supplement: Supplementary file 1 [file 40662_2026_518_MOESM1_ESM.docx]

**Supplementary Figure 1. Steady-state levels of glycolytic intermediates in proliferating and differentiated immortalized human meibomian gland epithelial cells.** The relative peak area of D-glucose 6-phosphate, fructose-6-phosphate, 2-phospho-D-glyceric acid and pyruvic acid were measured using ultra-performance liquid chromatography-tandem mass spectrometry (UPLC-MS/MS)-based non-targeted metabolomics. No significant differences were observed in the concentrations of these metabolites between the two cell states. (ns, *P* > 0.05, n = 3 independent biological replicates).
